# Association between dietary fat and fat subtypes with the risk of breast cancer in an Iranian population: a case-control study

**DOI:** 10.1186/s12944-021-01557-y

**Published:** 2021-10-17

**Authors:** Maedeh Mozafarinia, Bahareh Sasanfar, Fatemeh Toorang, Amin Salehi-Abargouei, Kazem Zendehdel

**Affiliations:** 1 Cancer Research Center, Cancer Institute of Iran, Tehran University of Medical Sciences, Tehran, Iran; 2 Nutrition and Food Security Research Center, Shahid Sadoughi University of Medical Sciences, Yazd, Iran; 3 Department of Nutrition, School of Public Health, Shahid Sadoughi University of Medical Sciences, Yazd, Iran; 4 Department of Community Nutrition, School of Nutritional Sciences and Dietetics, Tehran University of Medical Sciences, Tehran, Iran; 5 Cancer Biology Research Center, Cancer Institute of Iran, Tehran University of Medical Sciences, I.R. Tehran, Iran; 6 Breast Diseases Research Center, Cancer Institute of Iran, Tehran University of Medical Sciences, I.R. Tehran, Iran; 7 Cancer Research Center, Cancer Institute of Iran, Tehran University of Medical Sciences, P.O.Box 13145158, I.R. Tehran, Iran

**Keywords:** Fatty acids, Dietary fat, Polyunsaturated fatty acid, Animal fat saturated fatty acid, Monounsaturated fatty acid, Breast cancer, Neoplasms, Postmenopausal, Premenopausal

## Abstract

**Aim:**

To examine the relationship between dietary fat intake and breast cancer (BC) development.

**Method:**

This case-control study included 473 women with breast cancer (pathologically confirmed) and 501 healthy subjects matched by age and residency. Dietary intakes of different types and sources of fatty acids were assessed using a validated food frequency questionnaire. The association between dietary fats and odds of BC was assessed using a logistic regression model in crude and multivariable-adjusted models. *P* values below 0.05 were regarded as statistically significant.

**Results:**

Participants’ age and body mass index were 44.0 ± 10.8 years and 28.4 ± 5.6 kg/m^2^, respectively. Individuals with the highest quartile of total fat intake and polyunsaturated fatty acid (PUFA) intake were 1.50 times more at risk to develop BC than others. A positive significant association was observed between animal fat (Q4 vs. Q1, OR = 1.89, 95 % CI = 0.93–3.81), saturated fatty acid (SFA) (Q4 vs. Q1, OR = 1.70, 95 % CI = 0.88–3.30), monounsaturated fatty acid (MUFA) (Q4 vs. Q1 OR = 1.85, 95 % CI = 0.95–3.61) and PUFA intake (Q4 vs. Q1, OR = 2.12, 95 % CI = 1.05–4.27) with BC risk in postmenopausal women. However, there was no association in premenopausal women.

**Conclusions:**

Total dietary fat and its subtypes might increase the risk of BC, especially in postmenopausal women. This observational study confirms the role of dietary fat in breast cancer development. Intervention studies involving different estrogen receptor subgroups are needed.

**Supplementary Information:**

The online version contains supplementary material available at 10.1186/s12944-021-01557-y.

## Introduction

Breast cancer (BC) is the most prevalent cancer in women and the second foremost reason for cancer-related deaths in developed countries after lung cancer [1]. It is predicted that more than 2.1 million new BC cases occur worldwide annually, expressing over 24.2 % of malignancies in women [2]. Its incidence and mortality rates are higher in developed countries. The estimated Age Standard Rate (ASR) for breast cancer is estimated to be 35.8 per 100,000 women in 2020 and the disease is the third cause of death among Iranian women [3].

In addition to age, genetic and reproductive factors, other determinants like overweight or obesity, lack of physical activity, using alcohol and smoking as modifiable risk factors have been linked to BC [4, 5]. The role of diet has been shown as an important contributing factor to this condition. Dietary fat intake has long been hypothesized to increase BC risk; however, the findings were discrepant up to now [6, 7]. Some studies have only examined the total fat intake in association with BC risk, while others performed distinct analyses based on main fat subtypes. Some observational studies have shown a weak [8–13] or no significant association [14–18] between high fat intake and the risk of BC. Similar to these findings, no correlation was reported between n-3 and n-6 polyunsaturated (PUFA) fat intake and BC risk among Chinese women [19]. In contrast, a positive association was observed in some studies [10, 20–22]. Also, two studies [14, 15] found no association for eicosapentaenoic acid (EPA) and docosahexaenoic acid (DHA) as the two key n-3 PUFAs. However, Ouldamer et al. revealed that high dietary intake of EPA and DHA is associated with a 25 % decrease in BC risk [16]. Two case-control studies showed that animal fat intake was not associated with BC risk in premenopausal [17] and postmenopausal [18] women; however, significant associations were reported only among premenopausal women in some other observational studies [23–25].

Overall, a few studies have assessed the association between various types of fat intake and BC risk in the Middle-Eastern countries, as dietary intake and environmental issues are considerably different in this region (high amounts of carbohydrates, refined grains, or animal fats) [26]. Therefore, we aimed to examine if dietary total fat, animal fat, and different fatty acids intake are associated with the risk of BC in Iranian women.

## Methods and materials

### Study design and population

This was a case-control study performed from May 2014 to April 2016. Women aged 19–80 years (n = 486) with BC verified by pathological study entered the trial. All cases were recruited from patients referred to the Cancer Institute, situated at Imam Khomeini Complex in Tehran. Participants did not have any long-term dietary restrictions and history of any other cancers whom was new breast cancer cases. Controls (n = 516) were selected from healthy women who were relatives and friends of patients without cancer who were referred to Imam Khomeini Hospital Complex, Tehran, Iran. Controls were matched regarding the place of residence (Tehran province, other provinces) and age (5-year groups). Cases and controls were selected based on convenience sampling. According to the study design, participants who had either no response to more than 70 items of the food frequency questionnaire (FFQ) or a reported total energy intake of more than 5500 or less than 800 kcal/d (n = 116) were excluded. Ultimately, 473 cases and 501 controls entered the final analysis. Written informed consent was obtained from all participants. The study protocol was approved by the Bioethics Committee of Tehran University of Medical Sciences, Tehran, Iran (Ethics code: 93-03-51-27113).

**Dietary intake assessment**. The usual intake of 168 food items in the last year was assessed using a semi-quantitative FFQ by interviewing trained investigators. The reliability and validity of this FFQ were investigated by comparing data from two similar FFQ completed one year apart according to previous investigations [27, 28]. The FFQ used in this study included foods that Iranians usually consume. Participants were interviewed by a trained dietitian to report their food consumption (daily, weekly, monthly, or yearly). They also asked all participants to report their food consumption available only in specific seasons. For each food item, the reported frequency of consumption was converted to frequency per day and was multiplied by the standard portion size (grams) using household measures [29] to calculate grams per day. Then the daily energy and nutrient intake were calculated using the United States Department of Agriculture (USDA) food composition database modified for Iranian foods [30]. The daily nutrient intakes from food items were summed up to calculate the total daily intakes.

**Assessment of other variables.** BMI was calculated as weight in kilograms divided by height in meters squared. Physical activity assessment was done through the Global Physical Activity Questionnaire (GPAQ) validated for adults [31]. This questionnaire includes 16 items that quantify an average weekly physical activity level. The World Health Organization (WHO) developed the GPAQ to estimate activities in a typical week among these four domains; sedentary lifestyle, job-related activities, recreation, sports, and transportation. The data were then analyzed using the GPAQ guide [32]. The duration and frequency of physical activity (MET-h/wk) over a typical week were recorded. Moreover, a face-to-face interview was performed and other information including marital status, family history, alcohol consumption or tobacco use, menarche age, pregnancy, and obstetrics history (hormone therapy, and contraceptive use, infertility, menopause, etc.) were recorded.

### Statistical analysis

Data were analyzed according to menopausal status or in all participants as a whole. Total energy intake was adjusted as a confounding factor using the residual method [33]. Thereafter, subjects were categorized according to quartiles of dietary fatty acid intake. ANOVA, t-test, or Chi^2^ test was used where appropriate. Also, multivariable logistic regression was performed to evaluate any correlation between dietary fat and fatty acid with the development of BC. In the first model, adjustments were made for energy intake and age. In the multivariable model, further adjustments were considered for cigarette smoking, physical activity, alcohol consumption, BC family history, marital status, educational level, parity, and BMI. The first quartile of fat intake was considered as the reference group. All the analyses were performed using STATA version 14 (State Corp.). P values < 0.05 were considered as statistically significant.

## Results

The study included 473 cases (309 pre- and 158 postmenopausal women) and 501 controls (326 pre- and 165 postmenopausal women). Participants’ characteristics are depicted in Table 1.

**Table 1:** Baseline characteristics of the study participants

	Case (*n* = 473)	Control (*n* = 501)	p-value
Age (years)	45.8 ± 10.3	43.9 ± 11.2	**0.002**
BMI (kg/m2)	28.0 ± 5.1	28.8 ± 6.0	**0.01**
Physical activity (MET-h/week)	22.7 ± 40.2	29.4 ± 43.9	**0.006**
Age at menarche (years)	13.0 ± 2.5	12.9 ± 2.7	0.28
Menopausal status (%)			
Premenopausal	309 (66.1)	326 (66.4)	0.94
Postmenopausal	158 (33.8)	165 (33.6)	
Educational level (%)			
Un university	394 (84.3)	411 (84.0)	0.89
University	73 (15.6)	78 (15.9)	
Marital status (%)			
Married	437 (93.7)	462 (94.2)	0.74
Unmarried/divorced/widowed	29 (6.2)	28 (5.7)	
Family history of breast cancer (%)	46 (9.8)	7 (1.4)	**< 0.001**
Oral contraceptive use (%)	244 (53.0)	259 (61.2)	**0.01**
Current smoker (%)	18 (3.8)	25 (5.1)	0.34
Alcohol use (%)	12 (2.5)	30 (5.9)	**0.008**
Postmenopausal hormone use (%)	2 (0.42)	10 (2.00)	**0.02**
Parity			
Nulliparous/missing	210 (44.1)	215 (42.9)	0.90
1	42 (8.8)	51 (10.1)	
2–3	149 (31.3)	155 (30.9)	
≥ 4	75 (15.7)	80 (15.9)	

χ2 Test for ordinal qualitative variables and t-test for continuous variables

Abbreviation: BMI, Body mass index

Patients with BC were older (45.8 *νs*. 43.9 years), had a family history of BC more frequently (46 *νs*. 7 %), and had lower BMI (28.1 *νs*. 28.8 kg/m2) compared with the control subjects. Also, they had lower physical activity (22.7 *νs*. 29.4 MET h/wk). Besides, they were less likely to use oral contraceptives (53 vs. 61.2 %) postmenopausal hormones (0.42 vs. 2 %) or drink alcohol (2.5 vs. 5.9 %) than controls.

**Fig. 1:**
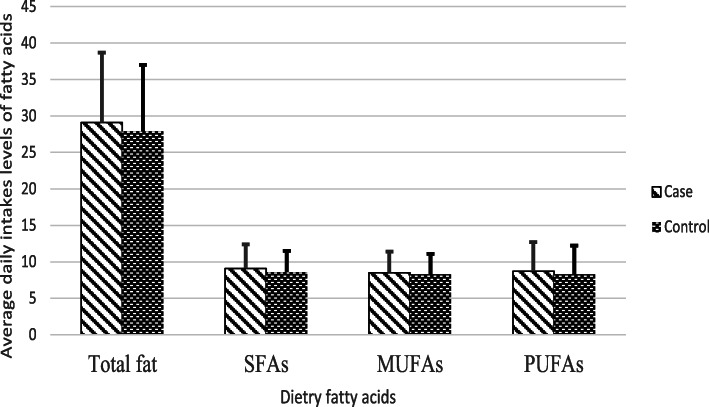
Mean (SD) energy adjusted values for dietary total fat, saturated (SFAs), mono-unsaturated (MUFAs) and poly-unsaturated (PUFAs) fatty acids in participants with and without breast cancer

As shown in Table 2, patients had a higher intake of total fat (21.9 vs. 27.9 g, *P* = 0.02), SFA (9.1 vs. 8.6 g, *P* < 0.01), and PUFAs (8.7 vs. 8.3, *P* = 0.05), and a lower intake of oleic acid (6.07 vs. 6.35, *P* = 0.05) compared with the controls **(**Fig. 1**)**. The stratification by menopausal status showed that premenopausal women with cancer had a significantly higher intake of energy (2769 vs. 2641 kcal/day, *P* = 0.05) and SFA (9.2 vs. 8.7 g, *P* = 0.01) than controls. Also, SFA (9 vs. 8.4 g, *P* = 0.04) intake was higher in postmenopausal women with cancer compared to controls.

**Table 2:** dietary fats intakes of the participants ^a^

	Case	Control				
	Mean ± SD	Mean ± SD		P value^a^		
**All women (473 case, 501 control)**						
Energy (kcal/d)	2673.3 ± 986.5	2619.7 ± 975.6		0.19		
Total fat	29.1 ± 9.6	27.9 ± 9.1		**0.02**		
Animal fat	22.6 ± 9.9	21.7 ± 9.0		0.06		
Vegetable fat	6.4 ± 4.0	6.2 ± 3.4		0.18		
Total SFA	9.1 ± 3.3	8.6 ± 2.9		**0.007**		
Myristic acid (14:0)	0.62 ± 0.34	0.63 ± 0.31		0.41		
Palmitic acid (16:0)	3.17 ± 1.1	3.19 ± 1.0		0.40		
Stearic acid (18:0)	1.23 ± 0.53	1.23 ± 0.49		0.44		
Total MUFA	8.5 ± 2.9	8.3 ± 2.8		0.17		
Palmitoleic acid (16:1n-7)	0.20 ± 0.098	0.21 ± 0.095		0.13		
Oleic acid (18:1n-9)	6.07 ± 2.7	6.35 ± 2.7		**0.05**		
Total PUFA	8.7 ± 4.0	8.3 ± 3.9		**0.05**		
n-3 PUFA						
EPA (20:5n-3)	0.011 ± 0.02	0.010 ± 0.01		0.25		
DPA (22:5n-3)	0.003 ± 0.003	0.003 ± 0.004		0.44		
DHA (22:6n-3)	0.01 ± 0.03	0.01 ± 0.02		0.35		
n-6 PUFA						
Linoleic acid (18:2n-6)	5.3 ± 2.9	5.1 ± 2.5		0.23		
Arachidonic acid (20:4n-6)	0.02 ± 0.01	0.03 ± 0.02		0.10		
Total Cholesterol	62.0 ± 30.6	62.9 ± 28.2		0.32		
**Premenopausal women (309 case, 326 control)**						
Energy (kcal/d)	2769.3 ± 990.5	2641.1 ± 995.6		**0.05**		
Total fat	29.3 ± 9.4	28.5 ± 9.1		0.12		
Animal fat	22.8 ± 9.8	22.0 ± 9.2		0.13		
Vegetable fat	6.5 ± 3.6	6.4 ± 3.7		0.47		
Total SFA	9.2 ± 3.2	8.7 ± 2.8		**0.01**		
Myristic acid (14:0)	0.63 ± 0.33	0.61 ± 0.29		0.28		
Palmitic acid (16:0)	3.2 ± 1.0	3.1 ± 0.99		0.44		
Stearic acid (18:0)	1.2 ± 0.52	1.2 ± 0.50		0.42		
Total MUFA	8.6 ± 2.9	8.5 ± 2.8		0.32		
Palmitoleic acid (16:1n-7)	0.20 ± 0.09	0.20 ± 0.09		0.48		
Oleic acid (18:1n-9)	6.1 ± 2.7	6.4 ± 2.8		0.07		
Total PUFA	8.8 ± 3.9	8.7 ± 3.9		0.33		
n-3 PUFA						
EPA (20:5n-3)	0.01 ± 0.02	0.01 ± 0.01		0.35		
DPA (22:5n-3)	0.003 ± 0.003	0.003 ± 0.003		0.26		
DHA (22:6n-3)	0.01 ± 0.02	0.01 ± 0.01		0.38		
n-6 PUFA						
Linoleic acid (18:2n-6)	5.2 ± 2.7	5.3 ± 2.7		0.42		
Arachidonic acid (20:4n-6)	0.03 ± 0.02	0.03 ± 0.02		0.33		
Total Cholesterol	63.9 ± 29.8	63.3 ± 27.6		0.39		
**Postmenopausal (158 case, 165 control)**						
Energy	2545.6 ± 951.5	2583.1 ± 959.0		0.36		
Total fat	28.8 ± 9.9	26.6 ± 8.9		**0.01**		
Animal fat	22.5 ± 10.1	20.9 ± 8.9		0.06		
Vegetable fat	6.2 ± 4.3	5.7 ± 2.9		0.09		
Total SFA	9.0 ± 3.5	8.4 ± 3.1		**0.04**		
Myristic acid (14:0)	0.62 ± 0.36	0.66 ± 0.33		0.15		
Palmitic acid (16:0)	3.1 ± 1.1	3.1 ± 1.0		0.31		
Stearic acid (18:0)	1.16 ± 0.55	1.19 ± 0.48		0.32		
Total MUFA	8.5 ± 2.9	8.0 ± 2.8		0.09		
Palmitoleic acid (16:1n-7)	0.20 ± 0.1	0.22 ± 0.09		0.06		
Oleic acid (18:1n-9)	5.9 ± 2.7	6.1 ± 2.7		0.20		
Total PUFA	8.5 ± 4.1	7.5 ± 3.7		**0.01**		
n-3 PUFA						
EPA (20:5n-3)	0.01 ± 0.02	0.01 ± 0.02		0.25		
DPA (22:5n-3)	0.003 ± 0.003	0.003 ± 0.004		0.22		
DHA (22:6n-3)	0.022 ± 0.03	0.021 ± 0.02		0.34		
n-6 PUFA						
Linoleic acid (18:2n-6)	5.1 ± 2.8	4.7 ± 2.2		0.09		
Arachidonic acid (20:4n-6)	0.02 ± 0.01	0.03 ± 0.02		0.10		
Total Cholesterol	59.1 ± 32.1	61.8 ± 29.5		0.21		

^a^ All value were % energy intake, All quantities for fatty acids were reported as grams

**Fig. 2:**
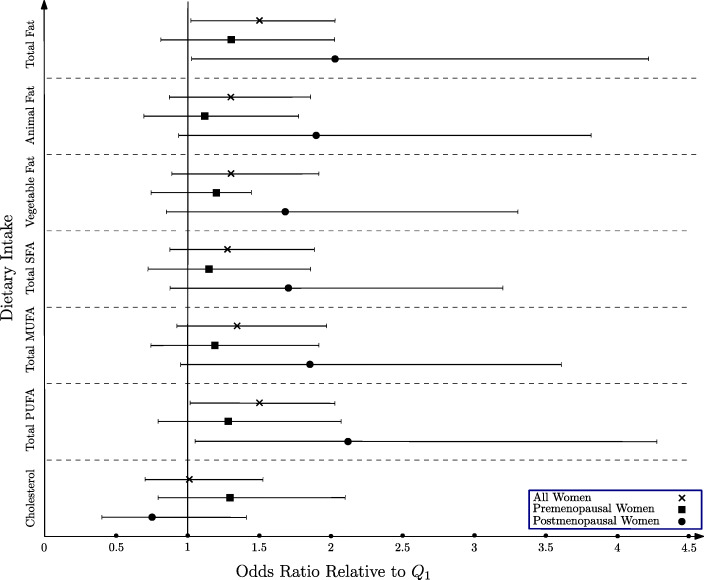
Odds ratios, with 95 % confidence intervals for all women, premenopause and postmenopause from multivariate logistic regression model of total fat, animal fat, vegetable fat, total SFA, total MUFA, total PUFA and cholesterol on women with and without breast cancer (cases, *n* = 473; controls, *n* = 501)

The estimated OR and 95 % CIs for BC according to quartiles of fat intake are shown in Table 3. Total fat intake was positively associated with the odds of BC in all participants as a whole. After confounding factors were controlled, women with highest total fat intakes had 1.50 times more risk to develop BC than those with lowest intakes. Besides, compared with women in the first and fourth quartiles, participants in the highest quartile of PUFA intake had a 1.50-time more risk to have BC (Fig. 2).

**Table 3:** Odds ratio (OR) and 95 % confidence intervals (CI) for breast cancer according to quartile of fat intake in women

	Quartile of intake				
	1	2	3	4	P value
	**Total fat**				
**All women**	107/126	105/124	125/125	133/126	
Age and energy adjusted OR	1	1.07 (0.72–1.57)	1.36 (0.92–2.01)	1.40 (0.97–2.02)	**0.03**
Multivariable OR	1	1.11 (0.74–1.67)	1.44 (0.95–2.16)	1.50 (1.02–2.20)	**0.01**
**Premenopausal women**	68/80	71/67	84/91	81/88	
Age and energy adjusted OR	1	1.46 (0.89–2.40)	1.40 (0.86–2.26)	1.27 (0.80-2.00)	0.4
Multivariable OR	1	1.53 (0.92–2.57)	1.43 (0.86–2.39)	1.31 (0.81–2.12)	0.36
**Postmenopausal women**	36/45	32/55	40/32	49/33	
Age and energy adjusted OR	1	0.66 (0.34–1.26)	1.40 (0.71–2.76)	1.88 (1.00-3.54)	**0.008**
Multivariable OR	1	0.66 (0.33–1.32)	1.62 (0.78–3.34)	2.16 (1.11–4.22)	**0.003**
	**Animal fat**				
**All women**	107/126	120/125	118/123	126/127	
Age and energy adjusted OR	1	1.19 (0.81–1.75)	1.28 (0.86–1.89)	1.28 (0.88–1.83)	0.19
Multivariable OR	1	1.10 (0.74–1.65)	1.38 (0.92–2.08)	1.28 (0.87–1.88)	0.12
**Premenopausal women**	70/84	76/76	78/73	81/93	
Age and energy adjusted OR	1	1.36 (0.84–2.19)	1.50 (0.92–2.46)	1.14 (0.73–1.78)	0.59
Multivariable OR	1	1.26 (0.76–2.08)	1.53 (0.91–2.57)	1.11 (0.69–1.77)	0.62
**Postmenopausal women**	33/41	42/48	39/45	43/31	
Age and energy adjusted OR	1	0.99 (0.52–1.88)	0.99 (0.50–1.92)	1.69 (0.87–3.28)	0.12
Multivariable OR	1	0.93 (0.47–1.86)	1.19 (0.59–2.42)	1.89 (0.93–3.81)	**0.05**
	**Vegetable fat**				
**All women**	119/126	142/123	85/123	130/129	
Age and energy adjusted OR	1	1.34 (0.92–1.95)	0.80 (0.53–1.22)	1.24 (0.86–1.78)	0.72
Multivariable OR	1	1.41 (0.95–2.08)	0.90 (0.59–1.39)	1.30 (0.89–1.91)	0.51
**Premenopausal women**	77/76	89/77	55/80	88/93	
Age and energy adjusted OR	1	1.30 (0.81–2.09)	0.83 (0.50–1.40)	1.13 (0.72–1.77)	0.97
Multivariable OR	1	1.45 (0.88–2.39)	0.92 (0.53–1.59)	1.20 (0.74–1.94)	0.87
**Postmenopausal women**	41/50	49/42	28/42	40/31	
Age and energy adjusted OR	1	1.34 (0.71–2.52)	0.73 (0.436–1.47)	1.56 (0.82–2.95)	0.45
Multivariable OR	1	1.29 (0.66–2.51)	0.82 (0.39–1.71)	1.68 (0.85–3.31)	0.29
	**Total SFA**				
**All women**	106/126	108/123	131/124	124/128	
Age and energy adjusted OR	1	1.08 (0.74–1.59)	1.46(0.99–2.14)	1.27 (0.88–1.83)	0.09
Multivariable OR	1	1.17 (0.78–1.74)	1.55 (1.04–2.32)	1.28 (0.87–1.88)	0.11
**Premenopausal women**	67/79	73/77	85/82	78/88	
Age and energy adjusted OR	1	1.15 (0.72–1.86)	1.53 (0.95–2.48)	1.17 (0.74–1.86)	0.34
Multivariable OR	1	1.26 (0.77–2.08)	1.50 (0.90–2.47)	1.14 (0.71–1.85)	0.52
**Postmenopausal women**	36/46	33/45	43/37	45/37	
Age and energy adjusted OR	1	0.87 (0.45–1.67)	1.40 (0.74–2.67)	1.56 (0.83–2.92)	0.07
Multivariable OR	1	0.96 (0.48–1.93)	1.75 (0.88–3.46)	1.70 (0.88–3.30)	**0.04**
	**Total MUFA**				
**All women**	109/126	114/124	120/124	131/127	
Age and energy adjusted OR	1	1.14 (0.78–1.68)	1.27 (0.86–1.88)	1.29 (0.90–1.86)	0.13
Multivariable OR	1	1.20 (0.81–1.79)	1.32 (0.88–1.98)	1.34 (0.92–1.96)	0.11
**Premenopausal women**	70/82	81/77	78/79	78/88	
Age and energy adjusted OR	1	1.48 (0.92–2.38)	1.56 (0.95–2.55)	1.17 (0.74–1.84)	0.55
Multivariable OR	1	1.51 (0.92–2.48)	1.53 (0.91–2.58)	1.19 (0.74–1.91)	0.57
**Postmenopausal women**	36/42	31/46	39/42	52/35	
Age and energy adjusted OR	1	0.68 (0.35–1.34)	0.98 (0.51–1.89)	1.70 (0.91–3.18)	**0.04**
Multivariable OR	1	0.74 (0.36–1.50)	1.09 (0.54–2.20)	1.85 (0.95–3.61)	**0.03**
	**Total PUFA**				
**All women**	98/126	121/123	130/124	123/128	
Age and energy adjusted OR	1	1.33 (0.9–1.95)	1.54 (1.05–2.26)	1.36 (0.94–1.97)	0.08
Multivariable OR	1	1.43 (0.96–2.14)	1.70 (1.13–2.55)	1.50 (1.02–2.22)	**0.03**
**Premenopausal women**	67/76	73/72	82/86	84/92	
Age and energy adjusted OR	1	1.29 (0.80–2.10)	1.31 (0.81–2.11)	1.16 (0.74–1.83)	0.56
Multivariable OR	1	1.45 (0.87–2.40)	1.48 (0.90–2.45)	1.28 (0.79–2.07)	0.37
**Postmenopausal women**	30/49	44/49	47/35	36/32	
Age and energy adjusted OR	1	1.42 (0.74–2.72)	2.11 (1.08–4.15)	1.90 (0.97–3.70)	**0.02**
Multivariable OR	1	1.41 (0.70–2.85)	2.42 (1.17–5.01)	2.12 (1.05–4.27)	**0.01**
	**Total Cholesterol**				
**All women**	132/126	110/124	117/125	116/126	
Age and energy adjusted OR	1	0.94 (0.65–1.36)	1.02 (0.70–1.49)	0.98 (0.68–1.40)	0.96
Multivariable OR	1	1.02 (0.69–1.51)	1.08 (0.72–1.60)	1.03 (0.71–1.52)	0.79
**Premenopausal women**	74/76	71/82	80/89	83/79	
Age and energy adjusted OR	1	1.04 (0.65–1.67)	1.12 (0.70–1.80)	1.26 (0.80–2.01)	0.27
Multivariable OR	1	1.09 (0.66–1.80)	1.19 (0.72–1.96)	1.29 (0.79–2.09)	0.27
**Postmenopausal women**	54/49	38/39	33/32	33/45	
Age and energy adjusted OR	1	0.82 (0.44–1.50)	0.89 (0.46–1.69)	0.65 (0.35–1.18)	0.20
Multivariable OR	1	1.02 (0.54–1.95)	0.91 (0.46–1.80)	0.75 (0.40–1.41)	0.35

Multivariable model: additionally, adjusted for cigar smoking, marital status, alcohol consumption, physical activity, education, family history of breast cancer, parity, and BMI

SFA: saturated fatty acids, MUFA: monounsaturated fatty acids, PUFA: polyunsaturated fatty acids

All quantities for fatty acids were reported as grams

In a subgroup analysis based on menopausal status, a positive association was found between animal fat (Q4 vs. Q1 OR = 1.89, 95 % CI = 0.93–3.81), SFA (Q4 vs. Q1 OR = 1.70, 95 % CI = 0.88–3.30), MUFA (Q4 vs. Q1 OR = 1.85, 95 % CI = 0.95–3.61) and PUFA intake (Q4 vs. Q1 OR = 2.12, 95 % CI = 1.05–4.27), with the risk of BC in postmenopausal women. Nonetheless, no association was found in premenopausal women.

## Discussion

The dietary total fat intake was positively associated with BC risk in all participants who entered the current investigation. Also, higher PUFA intake was related to an increased possibility of BC. However, the associations were significant only among postmenopausal women after the stratification of analyses based on menopausal status. These results indicate that a higher intake of animal fat, SFA, MUFA, and PUFA was associated with a higher risk of BC in postmenopausal women. Moreover, a direct association was observed between total fat and PUFA intake and BC risk in all participants as a whole. On the contrary, the Nurses’ Health Study found no association between any fat subtypes and BC [34]. Also, a meta-analysis reported no correlation between breast cancer risk and dietary total fat, SFA, MUFA, and PUFA intake [35]. This discrepancy might be due to different study populations or designs, different types of studied fatty acids, or possible measurement bias.

A significant positive association was also reported between dietary fat subtypes and the risk of BC in postmenopausal women, but there was no association in premenopausal women. In contrast to the current findings, two studies [23, 36] reported a significant positive association for animal fat in premenopausal women. Fatty acids composition of animal and vegetable origin are different, which might have varied effects on BC development [25]. In another analysis in premenopausal women, higher animal fat intake significantly predicted a higher mammographic density which in turn, increases the risk of BC [37]. However, in one retrospective study, hyperlipidemia was associated with higher breast density in premenopausal women [38].

In the present study, SFA and MUFA intake also increased BC risk in postmenopausal women. These findings regarding SFA were similar to some investigations that showed an increase in BC risk in postmenopausal women [39, 40]. However, Hunter, D. J. et al. reported no association between SFA intake and breast cancer after pooling 7 prospective cohorts [41]. It is proposed that a fat-rich diet is positively related to insulin resistance [42], which is probably involved in postmenopausal BC risk enhancement. Moreover, SFAs may increase the risk of insulin resistance and affect mammary tumorigenesis [43]. Besides, insulin resistance is associated with increased proinflammatory cytokines and decreased adiponectin levels, which could increase the probability of BC development in postmenopausal women [44]. A recent meta-analysis also revealed that low levels of adiponectin might increase BC risk in women experiencing menopause [45].

Moreover, in this study, there was a positive association between a higher intake of MUFA and the risk of BC in postmenopausal women. In line with the present study, in two observational studies, BC risk was directly correlated with MUFA intake [46, 47]. On the other hand, two other observational studies stated a protective effect of MUFA intake in BC development [48, 49]. These discrepancies may be due to the mechanisms triggered by oleic acid.

Stearoyl-CoA desaturase-1 (SCD1) enzyme is an important controller of fatty acid configuration in mammalian cells and plays a role in stearic acid to oleic acid conversion. In tumoral cells, SCD1 plays as a key regulator for lipogenesis able to enhance the activity of several oncogenic signaling pathways like Akt and PKC, which are activated by oleate [50]. Therefore, an association has been recently highlighted between SCD1 activity, MUFA, and tumor growth [51]. Another investigation in postmenopausal women reported that oleic acid levels might have a role in breast cancer development [52].

In the present investigation, higher PUFA intake was related to BC risk in women with menopause. Limited studies have examined the link between PUFA intake and BC risk stratified by menopausal status. However, in the Malmo Diet and Cancer study, Wirfalt. et al. reported a positive association between PUFA intake and BC development in postmenopausal women [10]. Also, one study suggested that higher PUFA (linoleic acid) intake might increase BC risk [48]. Furthermore, dietary intake of PUFA commonly includes high proportions of linoleic acid, which is a biosynthetic precursor of prostaglandin [53]. Arachidonic fatty acids and prostaglandin E2 could increase estrogen synthesis by inducing aromatase enzymes activity, which might affect cancer development [54]. Aromatase catalyzes the conversion of androgens to estrogens and vice versa [55]. Estradiol fatty acid esters can be accumulated in fat tissues at high levels [56]. The high levels of estradiol in the mammary tissues can induce estrogen receptor (ER) expression and influence cancer cell behavior [57]. There is some evidence that estrogen induces mammary cell proliferation by controlling the expression of some related genes [58, 59]. Moreover, linoleic acid has a role in T47D growth control. This effect is performed by alteration in the G13a G protein, estrogen receptor (ERa), or p38 MAP kinase gene expression [60, 61]. Also, Murillo-Ortiz et al. stated that increased levels of circulating estradiol could increase the risk of HR-positive BC in postmenopausal Mexican women [62]. Lowering serum estradiol levels by dietary prevention may still offer an approach to BC prevention [63].

Different associations found in pre- and post-menopausal women might be explained by differences in body fat percentage. As in this study, the hypothesis of associations between body fat and BC could not be approved in premenopausal women. Likewise, Zhao et al. in a study in premenopausal women reported that reduced expression of RPS6KB1, ESR1, and GATA3 in breast adipose tissue plays a role to decrease the risk of breast cancer [64]. Other researchers also concluded that altered expression of some genes (RRM2, SPP1, MMP9, Arf1) could be involved in increased cell proliferation of adipose tissue in breast cancer risks [65, 66]. However, in postmenopausal women, adipose fat is the essential source of circulating estrogen [63, 67]. In one previous study, dietary fat increased BC risk only in postmenopausal women [68]. Nonetheless, in some studies, there was no positive association between total fat or animal fat intake with estrogen levels in postmenopausal women [69, 70]. Further studies are recommended to elucidate the exact mechanisms.

### Study strengths and limitations

This investigation had some strengths. A quite big sample size, using standard questionnaires, performing a categorized analysis by menopausal status, and considering several confounding factors were some strengths. Prior studies found animal fat adversely affects BC risk in only premenopausal women; however, the current study found this association in postmenopausal women.

This study had some limitations. First, this was a case-control study, and the selection or recall bias could have affected the results. Second, the possibility of under- and over-reporting of either energy intake or special food groups and recall bias exists when using the semi-quantitative FFQ. Third, although FFQs are often used to categorize participants’ intakes rather than meticulous measurement of nutrient intake, a degree of gross misclassification is inevitable. Fourth, BC was not ruled out in controls. Fifth, the current study could not assess the hormonal receptors status and no information was available regarding breast cancer stage or grade.

## Conclusions

There was a direct association between total dietary fat and subtypes of fat intake with the risk of BC development particularly in women with menopause. This observational study provides support for the importance of dietary fat intake in increasing the risk of breast cancer. Therefore, the amount of dietary total fat as well as fatty acids should be recommended with caution to reduce the risk of BC. More intervention studies considering the effect of dietary fats on cell growth considering different subgroups of estrogen receptors are required.

## Supplementary information

**Additional file 1**

## Data Availability

The datasets generated and/or analyzed during the current study will be made available by request from the corresponding author.

## Abbreviations

BC: Breast cancer
BMI: Body Mass Index
DHA: Docosahexaenoic acid
EPA: Eicosapentaenoic acid
FFQ: food frequency questionnaire
GPAQ: Global Physical Activity Questionnaire
kcal/d: kilocalories per day
MET-minutes/weeks: Metabolic equivalent minutes per week
MUFA: Monounsaturated fatty acids
OR: Odds ratio
PUFA: Polyunsaturated fatty acids
SDs: standard deviations
SFAs: Saturated fatty acids
USDA: United States Department of Agriculture
WHO: World health organization
